# Circulating levels of AGEs and soluble RAGE isoforms are associated with all-cause mortality and development of cardiovascular complications in type 2 diabetes: a retrospective cohort study

**DOI:** 10.1186/s12933-022-01535-3

**Published:** 2022-06-06

**Authors:** Jacopo Sabbatinelli, Stefania Castiglione, Federica Macrì, Angelica Giuliani, Deborah Ramini, Maria Cristina Vinci, Elena Tortato, Anna Rita Bonfigli, Fabiola Olivieri, Angela Raucci

**Affiliations:** 1 Department of Clinical and Molecular Sciences, Università Politecnica delle Marche, Via Tronto 10/A, 60126 Ancona, Italy; 2 Laboratory Medicine Unit, Azienda Ospedaliero Universitaria “Ospedali Riuniti”, Ancona, Italy; 3 Experimental Cardio-Oncology and Cardiovascular Aging Unit, Centro Cardiologico Monzino-IRCCS, Milan, Italy; 4 Clinical Laboratory and Molecular Diagnostic, IRCCS INRCA, Ancona, Italy; 5 Unit of Vascular Biology and Regenerative Medicine, Centro Cardiologico Monzino-IRCCS, Milan, Italy; 6 Metabolic Diseases and Diabetology Department, IRCCS INRCA, Ancona, Italy; 7 Scientific Direction, IRCCS INRCA, Ancona, Italy

**Keywords:** Advanced glycation end-products, Biomarker, Major adverse cardiovascular events, Mortality, sRAGE, Type 2 diabetes

## Abstract

**Background:**

Advanced glycation end-products (AGEs) and their interaction with the receptor for advanced glycation end-products (RAGE) play a pivotal role in the development and progression of type 2 diabetes. In this retrospective cohort study, we explored the association of circulating levels of soluble RAGE (sRAGE) isoforms, i.e., endogenous secretory esRAGE and cleaved cRAGE, AGEs and their respective ratios with 15-year all-cause mortality in type 2 diabetes.

**Methods:**

Baseline AGEs and sRAGE isoforms concentration were measured by ELISA in 362 patients with type 2 diabetes and in 125 age- and gender-matched healthy control subjects (CTR). Independent predictors of mortality were determined using Cox proportional-hazards models and used to build and validate a nomogram for all-cause mortality prediction in type 2 diabetes.

**Results:**

AGEs, total sRAGE, cRAGE and the AGEs/sRAGE and AGEs/esRAGE ratios were significantly increased in patients with type 2 diabetes compared to CTR (p < 0.001). In CTR subjects, but not in type 2 diabetes patients, a significant negative correlation between cRAGE and age was confirmed (p = 0.003), whereas the AGEs/sRAGE (p = 0.032) and AGEs/cRAGE (p = 0.006) ratios were positively associated with age. At an average follow-up of 15 years (4,982 person-years), 130 deaths were observed. The increase in the AGEs/cRAGE ratio was accompanied by a higher risk of all-cause mortality in patients with type 2 diabetes (HR per each SD increment = 1.30, 95% CI 1.15–1.47; p < 0.001). Moreover, sRAGE was associated with the development of major adverse cardiovascular events (MACE) in type 2 diabetes patients without previous MACE (OR for each SD increase: 1.48, 95% CI 1.11–1.89). A nomogram based on age, sex, HbA1c, systolic blood pressure, and the AGEs/cRAGE ratio was built to predict 5-, 10- and 15-year survival in type 2 diabetes. Patients were categorized into quartiles of the monogram scores and Kaplan-Meier survival curves confirmed the prognostic accuracy of the model (log-rank p = 6.5 × 10^− 13^).

**Conclusions:**

The ratio between AGEs and the cRAGE isoform is predictive of 15-year survival in patients with type 2 diabetes. Our data support the assessment of circulating AGEs and soluble RAGE isoforms in patients with type 2 diabetes as predictors of MACE and all-cause mortality.

**Supplementary Information:**

The online version contains supplementary material available at 10.1186/s12933-022-01535-3.

## Background

Advanced glycation end products (AGEs) and the receptor for advanced glycation end products (RAGE) activate cell signaling pathways modulating inflammatory gene expression profile in several chronic inflammatory disorders [1–3]. AGEs are the products of non-enzymatic glycation and oxidation of proteins and lipids and under physiological conditions, anti-glycation defenses are sufficient to prevent their accumulation [2]. A significantly increased burden of AGEs was described in aging and age-related diseases (ARDs), primarily in diabetes mellitus [2, 4], and their levels, measured using skin autofluorescence, were associated with a significant increase in the incidence of major adverse cardiovascular events (MACE) in a multitude of chronic conditions, including heart failure [5], type 2 diabetes [6], and end-stage renal disease [7]. The contribution of AGEs in fostering endothelial dysfunction and exacerbating atherosclerosis has been confirmed by their robust association with non-invasive measures of arterial stiffness, which is more pronounced in men and younger individuals [8]. The membrane-bound RAGE (FL-RAGE) is a pattern recognition receptor that recognizes AGEs and several other ligands, some of which act as damage-associated molecular patterns (DAMPS) and pathogen-associated molecular patterns (PAMPs) [9–11]. Under physiological conditions, RAGE is expressed in the lungs while is almost undetectable in other tissues [12, 13]; however, its levels are induced by ligand accumulation [14–16]. The cytoplasmic domain of RAGE binds to the formin DIAPH1 that is essential for RAGE/ligand-mediated activation and production of reactive oxygen species (ROS) eventually promoting inflammatory processes in ARDs [17, 18].

The soluble forms of RAGE, collectively named (sRAGE), are also present in the blood and consist of the RAGE ectodomain, produced by proteolytic cleavage (cRAGE) of the membrane-bound form by metalloproteases or through alternative splicing as endogenous secretory RAGE (esRAGE), which is actively secreted [19]. sRAGE does not transduce signal upon ligand binding, acting as a decoy molecule to restrain the RAGE/ligand induced cell activation [20]. Accordingly, sRAGE administration reduces diabetes and atherosclerosis tissue remodeling, age-associated cardiac fibrosis and neointima expansion after vessel injury [13, 21–23].

In humans, altered circulating sRAGE and esRAGE levels have been related to disease states or their risk factors. Many studies reported lower sRAGE or esRAGE levels in subjects with cardiometabolic conditions like metabolic syndrome [24], hypertension [24, 25], obesity [26]and prediabetes [27–29]. sRAGE also decreases with aging in healthy subjects [30–32]. In contrast, sRAGE elevation has been associated with chronic diabetes [33, 34], frailty [35] and people with diminished kidney function [36].

The potential prognostic value of sRAGE as a marker of disease and the occurrence of adverse events seems to be suitable for individuals with chronic disease or multimorbidity and not for the general population [37–40]. Concerning diabetes, increased concentrations of sRAGE were associated with increased all-cause and cardiovascular (CV) mortality in type 1 diabetes [41, 42]. In patients with type 2 diabetes, higher levels of sRAGE were independently associated with new or worsening kidney disease and mortality over 5 years follow-up [43]. Prospective studies underlined significant positive association between sRAGE and esRAGE and the incident of coronary artery disease (CHD) in type 2 diabetes [38, 44].

Recently, the ratio of AGEs/sRAGE has been proposed as a more effective biomarker of organ damage than AGEs or sRAGE variants separately [31, 45, 46]. Moreover, a different predictive ability of esRAGE and cRAGE as markers of CV risk factors have been evidenced lately [29, 31, 32]. For instance, we have published that in a healthy population and long living individuals (LLIs), cRAGE negatively relates with aging while esRAGE is a more appropriate biomarker of obesity and longevity [31, 32]. Hence, new aspects should be taken in consideration in order to define the prognostic value of AGEs/sRAGE isoforms axis in human pathologies.

Herein, we simultaneously determined circulating levels of both sRAGE isoforms—esRAGE and cRAGE—AGEs and their respective ratios in patients with type 2 diabetes and in age- and gender-matched healthy control subjects and investigated their association with 15-year all-cause mortality in type 2 diabetes.

## Methods

### Samples

362 patients with type 2 diabetes and 125 age-and-gender matched healthy control subjects (CTRs) were randomly selected from a cohort of 562 patients with type 2 diabetes and 599 CTRs enrolled from central Italy [47]. The study was approved by the Institutional Review Board of IRCCS INRCA hospital (approval no. 34/CdB/03). Information such as vital signs, anthropometric measures, medical history, behavioral data including diet and physical activity were available for study participants. Written informed consent was obtained from each subject in accordance with principles of the Declaration of Helsinki. Type 2 diabetes was diagnosed according to the American Diabetes Association (ADA) criteria, i.e., patients having a glycated Hemoglobin A1c (HbA1C) ≥ 6.5% or fasting blood glucose ≥ 126 mg/dl or 2-hour blood glucose levels ≥ 200 mg/dl after the oral glucose tolerance test (OGTT), or a random blood glucose ≥ 200 mg/dl when severe diabetes symptoms are present [48]. Inclusion criteria for patients with diabetes were body mass index (BMI) < 40 kg/m^2^, age 40–87 years, ability, and willingness to give written informed consent. The presence/absence of diabetic complications was established as follows: diabetic retinopathy by fundoscopy through dilated pupils and/or fluorescence angiography; incipient nephropathy, defined as a urinary albumin excretion rate > 30 mg/24 h and a normal creatinine clearance; neuropathy established by electromyography; ischemic heart disease defined by clinical history, and/or ischemic electrocardiographic alterations; peripheral vascular disease, including atherosclerosis obliterans and cerebrovascular disease based on history, physical examinations and Doppler velocimetry. Among the 362 patients, 73 were affected by neuropathy, 27 by peripheral artery disease, 52 by diabetic kidney disease and 95 by retinopathy. Fifty-three patients had a history of major adverse cardiovascular events (MACE).

Fasting blood samples of all subjects were processed to obtain plasma and stored at − 80 °C.

### Measurement of RAGE isoforms and AGEs

Circulating levels of sRAGE isoforms were determined by commercial ELISA kits following manufacturer’s indications. Specifically, total human sRAGE included the detection of both cRAGE and esRAGE variants (DY1145, Human RAGE DuoSet ELISA, R&D Systems Inc., MN, USA) and esRAGE concentration was evaluated by an ELISA assay with an antibody raised against the exclusive C-terminal amino acids (332–347) sequence (K1009-1, B-bridge International, CA, USA). cRAGE was determined by subtracting esRAGE from sRAGE as already described [29, 31, 32].

AGEs were measured by an ELISA assay from Biolabs (STA-817 Cell Biolabs, INC. San Diego, CA) following manufacturer’s instructions. AGEs levels (µg/ml) were divided by sRAGE isoform levels (pg/ml) to obtain the ratio (µg/pg).

### Statistical analysis

Continuous variables were reported as either mean and standard deviation or median and interquartile range (IQR) based on their distribution (assessed using Shapiro-Wilk test). For comparisons between groups, Mann-Whitney U test and Kruskal-Wallis followed by Dunn post hoc test were used. Spearman correlation was used to assess correlations between continuous variables, which were visualized by correlation plots generated by the Jamovi software, version 2.2.1. Quantile regression was used to evaluate the impact of type 2 diabetes complications and treatments on the levels of AGEs and sRAGE isoforms. The association between AGEs and sRAGE isoforms and the follow-up endpoints was investigated by Cox proportional hazards analysis (adjusted for established risk factors and potential confounders) with 95% confidence intervals. Based on the results of the multivariate Cox regression analysis, a nomogram for predicting 5-, 10- and 15-year survival in type 2 diabetes was built using the “hdnom” package (version 6.0.0) for R, version 4.1 [49]. Specifically, a penalized Cox regression model trained with an adaptive elastic net procedure using 10-fold cross-validation on the whole sample. The adaptive elastic net is an adaptation of the original Cox regression for survival analysis which can handle correlated features and performs variable selection. Nomogram performance was assessed on 100 bootstrap samples. The predictive efficacy was assessed with calibration and discrimination statistics. Reclassification was assessed by use of the continuous net reclassification improvement (NRI^> 0^) [50].

## Results

Baseline demographical and biochemical characteristics of 362 patients affected by type 2 diabetes mellitus (median age 63.0 yrs., IQR 56.0–73.5 yrs) and 125 age- and gender-matched healthy control subjects (CTR; median age 67.0 yrs., IQR 60.0–72.0 yrs.) are reported in Table 1. Median type 2 diabetes duration was 12.5 yrs. (IQR 6.0–24.0 yrs.). No missing data were identified. The prevalence of diabetic complications was as follows: retinopathy, 26%; nephropathy, 15%; neuropathy, 20%; peripheral artery disease, 8%; MACE, 15%. Patients with type 2 diabetes showed significantly increased weight, BMI, waist-hip ratio (WHR), systolic (SBP) and diastolic (DBP) blood pressure, triglycerides, fasting glucose and insulin, HbA1C, HOMA index, creatinine, alanine aminotransferase, gamma-glutamyl transferase, white blood cells, hemoglobin, and high-sensitivity C-reactive protein (hs-CRP). On the contrary, total bilirubin and platelets were significant lower in type 2 diabetes compared to CTR. Moreover, serum levels of total cholesterol, HDL cholesterol (HDL-C), LDL cholesterol (LDL-C) and apolipoprotein A1 (ApoA1) were lower in patients, due to the higher prevalence of statin therapy compared to CTR.

**Table 1:** Baseline demographical and biochemical characteristics of healthy control subjects (CTR) and patients with type 2 diabetes

Variables	CTR (n = 125)	Type 2 diabetes (n = 362)	P
Age (years)	63.0 (56.0–73.5)	67.0 (60.0–72.0)	0.114
Gender (Males, %)	59 (47%)	200 (55%)	0.145
BMI (Kg/m^2^)	26.6 (24.0–29.2)	28.3 (25.9–31.4)	** < 0.001**
Weight (Kg)	73 (65–80)	78 (70–87)	** < 0.001**
Waist-hip ratio	0.90 (0.84–0.96)	0.94 (0.89–0.98)	** < 0.001**
Systolic blood pressure (mmHg)	133 (125–139)	136 (129–143)	** < 0.001**
Diastolic blood pressure (mmHg)	85 (80–93)	90 (85–96)	** < 0.001**
Total cholesterol (mg/dL)	217.5 (186.3–238.0)	209 (181.8–235.0)	0.124
HDL-C (mg/dL)	56.5 (47.3–67.8)	50.0 (43.0–60.0)	** < 0.001**
LDL-C (mg/dL)	128.2 (109.0–145.9)	116.6 (95.6–138.9)	**0.004**
Triglycerides (mg/dL)	92.5 (62.3–131.0)	115.0 (83.8–163.3)	** < 0.001**
ApoA1 (mg/dL)	176.0 (155.0–203.8)	164.5 (149.0–187.0)	**0.001**
ApoB (mg/dL)	101.5 (83.5–121.0)	101.0 (85.0–122.0)	0.822
Glucose (mg/dL)	94.0 (89.0–100.0)	152.0 (133.8–186.0)	** < 0.001**
HbA1C (%)	5.7 (5.5–6.1)	7.3 (6.5–8.1)	** < 0.001**
Insulin (UI/mL)	4.70 (3.44–6.96)	5.80 (3.69–8.83)	**0.003**
HOMA index	1.12 (0.77–1.61)	2.17 (1.42–3.61)	** < 0.001**
Creatinine (mg/dL)	0.85 (0.70–1.00)	0.90 (0.70–1.00)	**0.043**
eGFR (mL/min)	82.5 (68.3–89.5)	81.3 (66.1–87.5)	0.094
Azotemia (mg/dL)	38.0 (33.0–44.0)	38.0 (32.0–46.3)	0.706
Uric acid (mg/dL)	4.8 (4.1–5.5)	4.6 (4.0–5.4)	0.241
Alanine aminotransferase (U/L)	36 (32–42)	39 (33–48)	**0.002**
Aspartate aminotransferase (U/L)	21 (17–25)	20 (16–25)	0.104
Gamma-glutamiltransferase (U/L)	45 (35–56)	51 (41–62)	** < 0.001**
Total bilirubin (mg/dL)	0.7 (0.6–0.9)	0.6 (0.5–0.8)	**0.029**
WBC (n/mm^3^)	6.17 (5.09–7.29)	6.56 (5.60–7.62)	**0.003**
Hemoglobin (g/dL)	14.1 (13.4–15.0)	14.5 (13.6–15.4)	**0.033**
Platelets (n/mm^3^)	229 (195–273)	210 (176–251)	**0.008**
hs-CRP (mg/L)	1.98 (0.87–5.04)	2.73 (1.08–4.78)	**0.017**
PAI-1 (ng/mL)	17.8 (11.9–24.7)	18.5 (13.1–25.3)	0.674
Fibrinogen (mg/dL)	296 (249–369)	303 (257–344)	0.221
Iron (µg/dL)	79 (63–100)	81 (66–97)	0.375
Ferritin (ng/mL)	97.8 (49.0–187.3)	71.5 (39.6–150.7)	0.445
Disease duration (years)	–	12.5 (6.0–24.0)	–
Relevant medications (n, %)			
Any T2DM medication	–	264 (73%)	–
Metformin	–	133 (37%)	–
Sulphonylureas	–	173 (48%)	–
Glinides	–	7 (2%)	–
Insulin	–	60 (17%)	–
Vitamin K antagonists	–	39 (11%)	–
Statins	4 (3%)	60 (17%)	**< 0.001**
Type 2 diabetes complications (n, %)			
Any complication	–	194 (53%)	
Retinopathy	–	95 (26%)	–
Diabetic kidney disease	–	52 (15%)	–
Neuropathy	–	73 (20%)	–
Peripheral artery disease	–	27 (8%)	–
MACE	–	53 (15%)	–

Data are median (IQR) for continuous variables and n (%) for categorical variables. P value from Mann-Whitney test for continuous variables and from chi-squared tests of association for categorical variables. Significant differences are in bold

### Baseline assessment of AGEs and sRAGE isoforms

The comparisons of AGEs and the different isoforms of sRAGE between type 2 diabetes and CTR groups were reported in Table 2. AGEs as well as sRAGE levels were significantly increased in type 2 diabetes compared to CTR. The increase of sRAGE in type 2 diabetes mainly depends on cRAGE changes rather than esRAGE. Furthermore, the AGEs/sRAGE and AGEs/esRAGE ratios were increased in patients with type 2 diabetes while AGEs/cRAGE remained unchanged between the two groups. No significant gender-related differences were observed in both groups (data not shown).

**Table 2:** Comparison of AGEs, sRAGE isoforms, and their ratios between healthy controls (CTR) and patients with type 2 diabetes

	CTR (n = 125)	Type 2 diabetes (n = 362)	P-value
sRAGE (pg/mL)	430.6 (275.0–595.9)	667.7 (464.8–929.9)	** < 0.001**
esRAGE (pg/mL)	242.9 (153.9–362.3)	283.5 (138.0–432.9)	0.312
cRAGE (pg/mL)	149.0 (44.7–267.8)	324.1 (173.7–521.0)	** < 0.001**
AGEs (µg/ml)	4.41 (2.45–6.23)	11.15 (4.43–21.40)	** < 0.001**
AGEs/sRAGE	0.009 (0.005–0.018)	0.017 (0.006–0.035)	** < 0.001**
AGEs/esRAGE	0.016 (0.010–0.026)	0.055 (0.024–0.099)	** < 0.001**
AGEs/cRAGE	0.030 (0.011–0.103)	0.036 (0.011–0.095)	0.914

Data are median (IQR)

P-values for Mann–Whitney *U* test. Significant differences are in bold

**Fig. 1:**
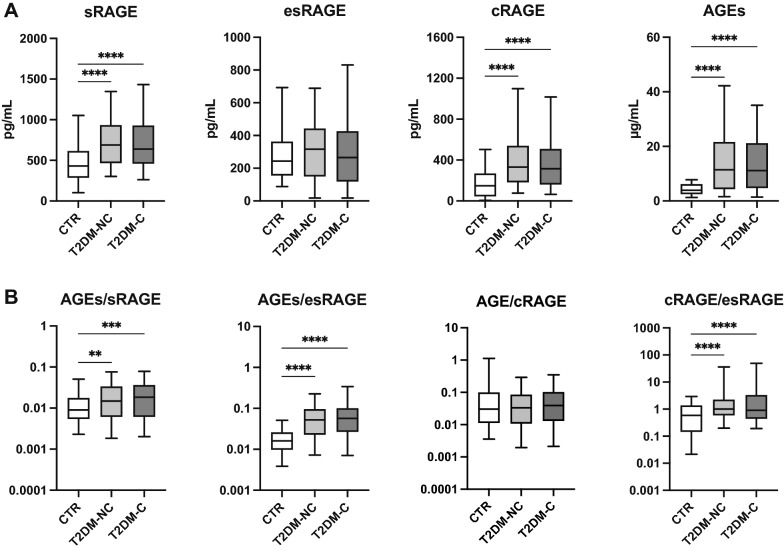
Boxplots for the comparison of **A** total sRAGE, esRAGE, and cRAGE isoforms and **)** AGEs/sRAGE, AGEs/esRAGE, AGEs/cRAGE, cRAGE/esRAGE ratio among healthy control subjects (CTR) and type 2 diabetes patients without (T2DM-NC) or with (T2DM-C) complications. **p < 0.01; ***p < 0.001; ****p < 0.0001 for Dunn’s post-hoc tests following Kruskal-Wallis *H* test

Figure 1 shows the results of the AGEs-sRAGE comparisons between CTR and patients with type 2 diabetes, grouped according to the absence (T2DM-NC) or presence (T2DM-C) of diabetic complications. While the differences between patients and CTR in AGEs, sRAGE, cRAGE, and the AGEs/sRAGE and AGEs/esRAGE ratios were confirmed, post-hoc analysis revealed no significant difference according to the presence of diabetic complications.

**Fig. 2:**
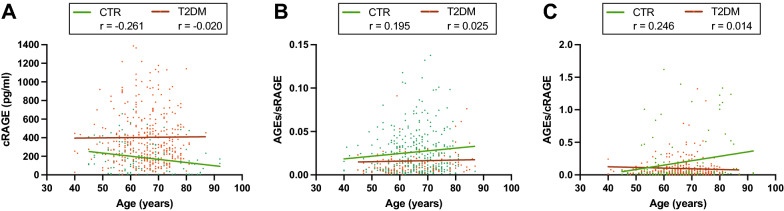
Scatter plot showing the correlation between subjects’ age and circulating **A** cRAGE, **B** AGEs/sRAGE and **C** AGEs/cRAGE ratios. Regression lines for CTR (green) and type 2 diabetes (T2DM, red) individuals are displayed

We previously showed that circulating levels of cRAGE, and not esRAGE, decline with aging in healthy subjects and LLIs [31, 32]. Table 3 shows the Spearman correlations of AGEs, the different isoforms of sRAGE and their ratios with age in CTR and type 2 diabetes groups. In CTR subjects, a significant negative correlation between cRAGE and age was confirmed [32]  (Fig. 2A), whereas the AGEs/sRAGE and AGEs/cRAGE ratios were positively associated with age (Fig. 2B, C), however, cRAGE and AGEs/cRAGE evidenced higher correlation coefficients, in terms of absolute values, than AGEs/sRAGE Fig. 2). No significant correlations were observed in the type 2 diabetes patient’s group. Table 3). Overall, these data indicate that cRAGE and AGEs/cRAGE ratio represent valuable biomarkers of chronological age in healthy subjects.

**Table 3:** Spearman correlations of AGEs and the different isoforms of sRAGE isoforms with age in healthy controls (CTR) and patients with type 2 diabetes

		CTR (n = 156)	Type 2 diabetes (n = 362)
sRAGE	Correlation coefficient	− 0.161	− 0.052
	P-value	0.073	0.323
esRAGE	Correlation coefficient	0.042	− 0.051
	P-value	0.646	0.330
cRAGE	Correlation coefficient	− 0.261**	− 0.020
	P-value	**0.003**	0.711
AGEs	Correlation coefficient	0.069	0.028
	P-value	0.446	0.600
AGEs/sRAGE	Correlation coefficient	0.195*	0.025
	P-value	**0.032**	0.631
AGEs/esRAGE	Correlation coefficient	0.035	0.081
	P-value	0.701	0.123
AGEs/cRAGE	Correlation coefficient	0.246**	0.014
	P-value	**0.006**	0.796

^**^p < 0.01 for Spearman’s regression. Significant correlations are in bold

**Fig. 3:**
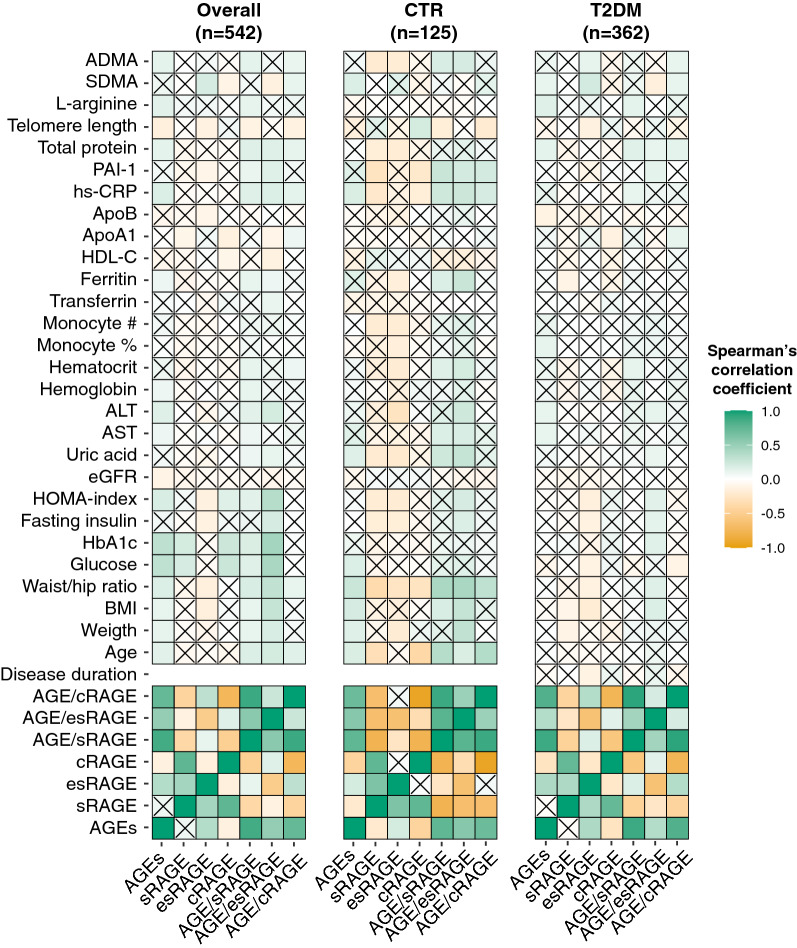
Correlation plots showing the correlations among the different variables and AGEs, sRAGE isoforms and the derived ratios in the whole population and in CTR and type 2 diabetes (T2DM) subjects separately. The intensity of the color depends on the magnitude of the Spearman’s correlation. Non-significant correlations (p ≥ 0.05) are crossed

Then, we explored the correlations between AGEs and sRAGE isoforms and the available biochemical variables. The correlation plots in Fig. 3 summarize the Spearman’s correlation coefficients for each pair of variables in the entire cohort and in CTR and type 2 diabetes subjects separately. Circulating levels of AGEs were positively associated to variables related to blood glucose control and insulin resistance, i.e., BMI, WHR, glucose, HbA1c and HOMA-index, in the entire cohort and in CTR subjects, but not in type 2 diabetes. Moreover, AGEs and sRAGE isoforms were variably associated with the levels of hs-CRP, plasminogen activator inhibitor-1 (PAI-1) and the endogenous endothelial nitric oxide synthase (eNOS) inhibitor ADMA and SDMA. Notably, in diabetes patients, esRAGE levels were negatively associated with the disease duration. The complete correlation matrix is available as Additional file 1: Table S1.

The levels of AGEs and sRAGE variants were analyzed according to the presence of the micro- and macrovascular complications of diabetes, i.e., neuropathy, nephropathy, retinopathy, peripheral vascular disease, and MACE (Table 4). Results of the quantile regression model, adjusted for age and HbA1c, revealed that nephropathy was associated with decreased sRAGE levels and increased AGEs/sRAGE and AGEs/esRAGE ratios. Retinopathy was associated with increased cRAGE and reduced AGEs levels, AGEs/sRAGE and AGEs/cRAGE ratios. The presence of atherosclerotic vascular disease was associated with increased cRAGE levels, while subjects with history of MACE showed a reduced AGEs/esRAGE ratio. Moreover, patients under insulin therapy were characterized by significantly reduced AGEs/sRAGE, AGEs/cRAGE and AGEs levels, after adjustment for age and HbA1c (Table 5).

**Table 4:** Age- and HbA1c-adjusted multiple quantile regression model for the evaluation of AGEs and sRAGE isoforms in type 2 diabetes complications

	Neuropathy		Nephropathy		Retinopathy		PAD		MACE	
Variable	Coeff.	p	Coeff.	p	Coeff.	p	Coeff.	p	Coeff.	p
sRAGE	− 130.7	**0.041**	2.130	0.976	44.7	0.456	26.4	0.786	− 9.875	0.889
cRAGE	− 30.40	0.475	28.49	0.551	85.38	**0.034**	127.9	**0.049**	− 81.14	0.087
AGEs	3.187	0.158	− 0.487	0.847	− 5.965	**0.005**	− 0.863	0.801	− 1.23	0.466
AGEs/sRAGE	0.010	**0.008**	0.001	0.846	− 0.009	**0.016**	− 0.005	0.349	− 0.001	0.794
AGEs/esRAGE	0.020	**0.038**	− 0.001	0.960	− 0.005	0.579	0.007	0.642	− 0.023	**0.028**
AGEs/cRAGE	0.016	0.090	0.005	0.656	− 0.024	**0.006**	− 0.013	0.363	0.001	0.912

In bold significant associations

Quantile regression models the relationship between a set of predictor variables (type 2 diabetes complications) and specific quantiles of target variables

Coefficient are displayed for the 0.5 quantile (median)

**Table 5:** Age- and HbA1c-adjusted multiple quantile regression model for the evaluation of AGEs and sRAGE isoforms according to type 2 diabetes treatments

	Metformin		Sulphonylureas		Glinides		Insulin	
Variable	Coeff.	p	Coeff.	p	Coeff.	p	Coeff.	p
AGEs	− 1.462	0.439	− 2.088	0.262	0.992	0.880	− 5.060	**0.050**
AGEs/sRAGE	0.002	0.532	− 0.002	0.487	0.014	0.180	− 0.008	**0.048**
AGEs/cRAGE	0.002	0.767	− 0.003	0.700	0.034	0.231	− 0.026	**0.021**

In bold significant associations

Coefficient are displayed for the 0.5 quantile (median)

### Prognostic value of sRAGE isoforms in the follow-up of type 2 diabetes

After 15 years of follow-up (4,982 person-years), 130 out of 352 patients with type 2 diabetes were deceased (36.9%). The observed crude mortality was 26.1 per 1000 person-years. Mean survival was longer in patients without complications compared to patients with at least one complication (181.0 [173.9–188.1] vs. 160.8 [152.8–168.9] months, p < 0.001).

Cox regression models were applied to identify association between AGEs and sRAGE isoform levels and all-cause mortality in type 2 diabetes. Table 6 shows the univariate HRs for the established all-cause mortality risk predictors—age, sex, BMI, disease duration, SBP, HbA1c, blood lipids, eGFR, hs-CRP, and ongoing treatments—and for AGEs, sRAGE isoforms, and their ratios. Cox regression univariate and multivariate analyses, adjusted for the abovementioned confounders, revealed an increased mortality risk for patients with increased AGEs/cRAGE ratio **(**Table 6**)**. The combined model, which includes the established risk factors and the ratios between AGEs and sRAGE isoforms as predictors, confirmed that each SD increase in the AGEs/cRAGE ratio is accompanied by a higher risk of mortality in type 2 diabetes (HR: 1.30, 95% CI 1.15–1.47; Table 6).

**Table 6:** Univariate and multivariate Cox regression analysis for the prediction of 15-year all-cause mortality in patients with type 2 diabetes

Model	Univariate		Multivariate		Combined model	
Predictor	HR (95% CI)	p	HR (95% CI)	p	HR (95% CI)	p
Established risk factors						
Sex (male)	1.20 (0.83–1.74)	0.329	–	–	1.47 (0.94–2.30)	0.093
Age (years)	1.11 (1.08–1.14)	< 0.001	–	–	**1.10 (1.07–1.14)**	** < 0.001**
Disease duration (years)	1.03 (1.01–1.04)	< 0.001	–	–	**1.02 (1.01–1.04)**	**0.008**
BMI (Kg/m^2^)	1.01 (0.97–1.05)	0.593	–	–	1.01 (0.96–1.06)	0.647
SBP (10 mmHg increase)	1.48 (1.24–1.77)	< 0.001	–	–	**1.37 (1.12–1.68)**	**0.002**
HbA1c (%)	1.09 (0.95–1.24)	0.217	–	–	1.10 (0.93–1.30)	0.255
Total cholesterol (SD-increase)	0.95 (0.79–1.14)	0.552	–	–	1.21 (0.74–1.96)	0.445
LDL-C (SD-increase)	0.82 (0.68–0.99)	0.034	–	–	0.75 (0.50–1.14)	0.179
HDL-C (SD–increase)	0.92 (0.75–1.13)	0.427	–	–	0.88 (0.65–1.20)	0.433
Triglycerides (SD-increase)	1.37 (1.10–1.71)	0.005	–	–	1.15 (0.76–1.75)	0.514
eGFR (10 mL/min increase)	0.78 (0.72–0.86)	< 0.001	–	–	0.94 (0.86–1.03)	0.192
hs-CRP (SD-increase)	1.14 (1.00–1.31)	0.052	–	–	**1.18 (1.01–1.39)**	**0.036**
Relevant treatments			–	–		
Insulin	1.66 (1.08–2.54)	0.020	–	–	0.89 (0.51–1.55)	0.675
Metformin	0.68 (0.46–1.01)	0.058	–	–	**0.63 (0.41–0.97)**	**0.037**
Sulphonylureas	1.10 (0.76–1.58)	0.624	–	–	0.81 (0.53–1.23)	0.324
Statins	1.18 (0.75–1.87)	0.468	–	–	0.93 (0.56–1.56)	0.785
Vitamin K antagonists	1.57 (0.94–2.62)	0.088	–	–	1.29 (0.74–2.26)	0.376
Candidate predictors (SD-increment)						
sRAGE	0.89 (0.74–1.07)	0.225	–	–	–	–
esRAGE	0.93 (0.77–1.13)	0.469	–	–	–	–
cRAGE	0.93 (0.77–1.12)	0.436	–	–	–	–
AGEs	0.88 (0.70–1.10)	0.249	–	–	–	–
AGEs/sRAGE	0.90 (0.73 –1.11)	0.326	0.86 (0.69–1.08)	0.203	0.79 (0.61–1.02)	0.072
AGEs/esRAGE	0.97 (0.77–1.21)	0.760	0.84 (0.66–1.07)	0.159	0.88 (0.71–1.11)	0.289
AGEs/cRAGE	1.20 (1.06–1.35)	0.005	**1.24 (1.07–1.43)**	**0.004**	**1.30 (1.15–1.47)**	** < 0.001**
cRAGEs/esRAGE	0.95 (0.78–1.16)	0.629	0.84 (0.69 –1.02)	0.080	–	–

In the multivariate model, each ratio is adjusted for established risk factors and relevant treatments. Combined model adjusted for AGEs/sRAGE, AGEs/esRAGE, AGEs/cRAGE, established risk factors and relevant treatments. Significant predictors at the multivariate analysis are displayed in bold

**Fig. 4:**
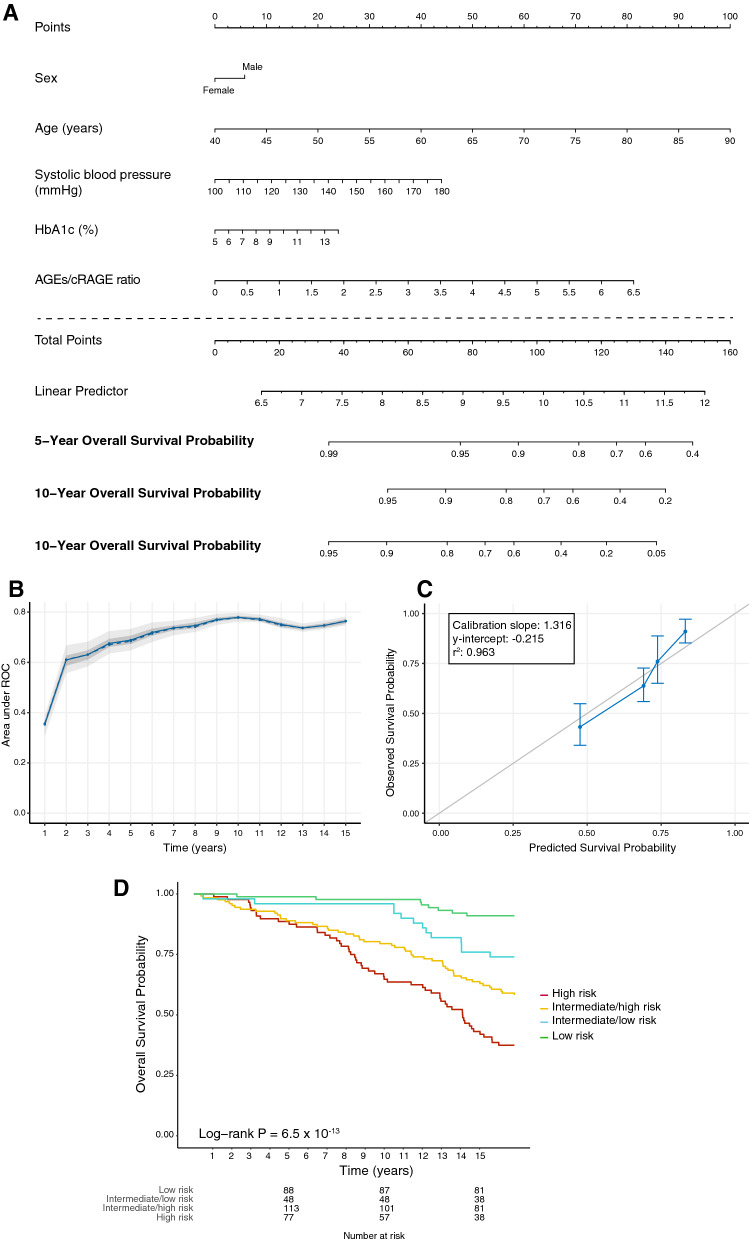
**A** Nomogram for predicting 5-, 10-, and 15-year overall survival in patients with type 2 diabetes based on sex, age, systolic blood pressure (mmHg), HbA1c (%), and the AGEs/cRAGE ratio. The points assigned to each variable are summed up to obtain the total points, and a vertical line can be drawn from the total points to obtain the corresponding survival probability. **B** Performance of the model based on the internal validation. Model area under the curves (AUCs) at each year are displayed. The solid line represents the mean of the AUC, the dashed line represents the median of the AUC. The darker interval in the plot shows the 25% and 75% quantiles of AUC, the lighter interval shows the minimum and maximum of AUC. **C** Plot of the observed against the predicted 15-year survival probability for patients with type 2 diabetes grouped according to risk quartiles. The grey diagonal line represents perfect calibration. **D** Kaplan-Meier survival function for patients with type 2 diabetes grouped according to quartiles of the nomogram-based mortality risk score

Based on the results of the Cox regression models, an adaptive elastic net regression model and multivariate Cox regression analysis was computed to predict survival at 5, 10 and 15 years in type 2 diabetes based on the following variables: age, disease duration, sex, HbA1c, SBP, hs-CRP, and the AGEs/cRAGE ratio. Disease duration and hs-CRP were excluded from the model by the elastic-net regression process. Figure 4A presents the model in the form of a nomogram that provides the long-term survival probabilities corresponding to a particular total score. The total score for a patient is obtained by adding up the scores for each of the six predictors. Internal validation with bootstrap resampling performed on the whole sample showed that the AUCs for the all-cause mortality prediction nomogram were 0.688, 0.779 and 0.764 at 5, 10 and 15 years respectively (Fig. 4B). The internal calibration plot revealed a good agreement between the observed and predicted values (calibration slope 1.32, y-intercept = − 0,22 [95% CI − 0.76 to 0.33]; Fig. 4C). Based on the risk profile predicted by the nomogram, 4 homogeneous groups of patients were generated, and Kaplan-Meier survival curves were constructed (Fig. 4D). The log-rank test confirmed that the survival curves of patients grouped according to the nomogram-based mortality risk score were significantly different (p = 6.5 × 10^− 13^).

AGEs and the sRAGE isoforms were also evaluated as predictors of the development of diabetic complications. While none of the parameters was significantly associated with development of any complication in patients without complications at the time of enrolment, sRAGE was associated with the development of MACE over a 15-year follow-up in patients with type 2 diabetes who had no history of MACE at recruitment (OR for each SD-increase: 1.48, 95% CI 1.11–1.98; Additional file 1: Table S2).

Finally, we tested whether the AGEs/cRAGE ratio added significantly to the 10-year all-cause mortality risk predicted by the RECODe equation [51]. The term related to urinary albumin-to-creatinine ratio (UACR), which was not assessed at the time of recruitment, has been omitted from the equation, as recommended by the original publication. The 10-year probability of all-cause mortality in our cohort ranged from 1.9 to 76.9%, with a median of 13.6%. The category-free, continuous NRI^> 0^ of the predictive model obtained by adding the AGEs/cRAGE ratio to the 10-year all-cause mortality probability predicted by the RECODe equation was 0.090 (95% CI, 0.020–0.351) at 10 years of follow-up.

## Discussion

This study compared levels of soluble RAGE isoforms, AGEs, and their respective ratios in type 2 diabetes patients, with an average disease duration of 12.5 years, and age- and sex-matched healthy individuals, and explored their ability to predict all-cause mortality and development of MACE in patients with type 2 diabetes. Our results showed that circulating AGEs and sRAGE levels are increased in type 2 diabetes and that the increase of sRAGE is due to cRAGE increment since esRAGE did not change. Accordingly, the AGEs/sRAGE and AGEs/esRAGE ratios were also increased in type 2 diabetes. Moreover, we demonstrated that the AGEs/cRAGE ratio was able to predict all-cause mortality and that sRAGE was associated with the development of MACE in patients with type 2 diabetes. Finally, we built and internally validated a nomogram for the prediction of long-term survival probability in type 2 diabetes based on age, sex, HbA1c, SBP, and the AGE/cRAGE ratio.

Although several studies reported a significant increase in AGE levels in patients with type 2 diabetes [43, 52, 53], more conflicting evidence are available on sRAGE. Most cross-sectional studies focused on total sRAGE levels, with findings variable on type of diabetes, disease duration and presence of complications [54–57]. A smaller number of studies assessed more in-depth the sRAGE isoforms, with no conclusive results [29, 53]. A recent meta-analysis showed that circulating esRAGE was lower and inversely correlated with carotid intima-media thickness (IMT) in type 2 diabetes whereas a contrasting relationship was described between sRAGE and carotid IMT in patients with type 1 diabetes [58]. Overall, there is consensus that sRAGE levels reflect the extent of RAGE overexpression observed in immune [59], endothelial [60] and vascular smooth cells [61] in patients and animal models of diabetes. Our data support the evidence that the induction of FL-RAGE expression and its constitutive shedding by ADAM10 is responsible for the increment of circulating cRAGE in type 2 diabetes, however a concomitant increase of ADAM10 expression and activity, already observed in type 1 diabetes, cannot be excluded [62].

Here, we reported multiple associations between the presence of diabetic complications and AGEs, sRAGE isoforms and their ratios. In agreement with a previous study reporting an association between esRAGE and the severity of coronary artery disease in patients with type 2 diabetes [63], we observed a lower AGEs/esRAGE ratio in patients with a history of MACE. The strongest patterns of correlation were, however, observed for diabetic neuropathy and retinopathy. Indeed, the former was accompanied by lower levels of sRAGE (and higher AGEs/sRAGE ratio), while lower AGEs and increased cRAGE levels were observed in the latter. The interpretation of these apparently contrasting observations could be limited by the presence of a number of undiagnosed microvascular complications and might be related to the high degree of heterogeneity in terms of disease duration among patients with different complications. Indeed, it has been hypothesized that the shape of the relationship between AGEs and sRAGE isoforms is modelled according to the severity of type 2 diabetes complications, reflecting the progressive exhaustion of the sRAGE compensatory mechanism against AGEs [64].

Interestingly, we observed that AGEs/sRAGE, AGEs/cRAGE and AGEs levels were significantly lower in patients treated with insulin independent from HbA1c. Our findings are consistent with the in vitro observation that insulin not only increases both FL-RAGE and esRAGE expression but can also stimulate the shedding of cRAGE from the membrane-bound receptor [65].

Recently, we explored the role of circulating sRAGE isoforms in healthy human aging. When we previously determined serum concentration of both isoforms of sRAGE, esRAGE and cRAGE, and their ligands AGEs, HMGB1 and S100A8/A9 in a healthy population ranging from 20 to 90 years, we observed that cRAGE showed a negative correlation with age while RAGE ligands – AGEs and S100A8/A9 – increased with advancing age [32]. This result was confirmed in a recent study including LLIs, i.e., subjects older than 90 years [31]. Overall, LLIs are characterized by a lower AGEs/sRAGE ratio, due to esRAGE increase and AGEs reduction which may explain their reduced cardiovascular and metabolic risk [31]. Hence, we can assume that circulating cRAGE could be considered a reliable marker of chronological age, while esRAGE a protective factor associated with longevity.

The prognostic value of measuring sRAGE levels in blood is a matter of debate. Findings from the ADVANCE [43] and CARD [38] studies demonstrated that increased levels of sRAGE in patients with type 2 diabetes are independent predictors of new-onset or worsening renal disease, incident coronary heart disease and all-cause mortality within 5 years, without providing additional information on the specific isoforms. Here, we show for the first time that a predictive model encompassing age, sex, HbA1c, SBP, and the AGEs/cRAGE ratio was able to stratify patients with type 2 diabetes according to the all-cause mortality risk. The model performed better for > 5 y follow-up time span, reaching its maximum predictive value at 10 years. Our results further corroborate the significance of the assessment of AGEs and their circulating receptors in type 2 diabetes and provide added value for a more comprehensive evaluation of sRAGE isoforms, also in relation to AGE levels. In this regard, it has been demonstrated that sRAGE acts as a decoy receptor by preventing ligands from interacting with membrane-bound FL-RAGE [66]. While no agreement has been reached on the sRAGE isoform concentrations required to scavenge circulating ligands [39], here we provide clues that a disproportionate presence of AGEs not adequately counterbalanced by the cRAGE isoform is associated with reduced survival in type 2 diabetes. Our results uncovered a complex pattern of association between circulating sRAGE isoforms and AGEs in determining the risk of mortality, which supports the growing consensus that the ratios between AGEs and sRAGE isoforms may be more informative for the prediction of type 2 diabetes clinical course rather than sRAGE alone. Notably, the AGEs/cRAGE ratio led to a significant, albeit modest, improvement in the ability of the already established RECODe model of predicting 10-year all-cause mortality in type 2 diabetes based on age, sex, ethnicity, smoking, SBP, history of MACE, HbA1c, total cholesterol, HDL-C, serum creatinine, and UACR [51]. Furthermore, we observed that each SD-increase in plasma sRAGE was independently associated with the development of MACE during a > 15-years follow-up in type 2 diabetes patients with no history of MACE at recruitment. Our findings are consistent with previous reports showing that serum sRAGE levels were independently associated with CV outcomes in type 2 [38, 44] and type 1 diabetes [41].

A number of limitations should be acknowledged. First, reliance on a single baseline measurement for AGEs and sRAGE isoforms could have introduced an additional bias due to biological and analytical variability. Second, due to the unavailability of data regarding the mortality of control subjects we were unable to estimate the excess mortality in diabetes due to the increase of circulating AGEs and sRAGE. Third, the lack of data on the UACR at the time of recruitment prevented us to calculate the full-term RECODe equation for the 10-year all-cause mortality. However, we believe that our results were minimally affected by this shortcoming, given the relatively small impact of UACR on the equation results (β = 0.00039) and the proven robustness of the equation in presence of missing data. Moreover, external validation of the nomogram was not performed due to the relatively small sample size, thus limiting the generalization of our findings. Finally, the high heterogeneity of diabetes phenotypes in our cohort prevented us to draw definitive conclusions on the modulation of these biomarkers according to specific complications and treatments but allowed us to perform a more comprehensive and accurate analysis of their prognostic role in type 2 diabetes.

## Conclusions

In conclusion, our data support the assessment of circulating AGEs and soluble RAGE isoforms in patients with type 2 diabetes as predictors of MACE and all-cause mortality. Further multicenter external validation studies should be performed to verify the prognostic value and generalizability of our nomogram.

## Supplementary Information

**Additional file 1:** **Table S1.** Correlation matrix between selected clinical/biochemical variables and plasma levels of AGEs and sRAGE isoforms in CTR (n = 125) and T2DM (n = 362) subjects. **Table S2.** Binary logistic regression for the prediction of the development of MACE in patients with T2DM and no history of MACE at recruitment.

## Data Availability

The datasets generated during and/or analyzed during the current study are available from the corresponding author on reasonable request.

## Abbreviations

ADA: American Diabetes Association
AGEs: Advanced glycation end-products
ApoA1: Apolipoprotein A1
ARDs: Age-related diseases
AUC: Area Under the Curve
BMI: Body mass index
CHD: Coronary artery disease
CI: Confidence interval
cRAGE: Cleaved RAGE
CTR: Control subjects
CV: Cardiovascular
CVD: Cardiovascular disease
DAMPS: Damage-associated molecular patterns
DBP: Diastolic blood pressure
eNOS: Endothelial nitric oxide synthase
esRAGE: Endogenous secretory
FL-RAGE: Membrane-bound RAGE
HbA1c: Hemoglobin A1c
HDL-C: HDL cholesterol
HOMA: Homeostatic model assessment
HR: Hazard ratio
hs-CRP: High sensitivity C Reactive Protein
IMT: Intima-media thickness
IQR: Interquartile range
LDL-C: LDL cholesterol
LLIs: Long living individuals
MACE: Major adverse cardiac events
OGTT: Oral glucose tolerance test
PAI-1: Plasminogen activator inhibitor-1
PAMPs: Pathogen-associated molecular pattern
RAGE: Receptor for advanced glycation end-products
ROS: Reactive oxygen species
SBP: Systolic blood pressure
sRAGE: Soluble RAGE
T2DM-C: Type 2 diabetes with complications
T2DM-NC: Type 2 diabetes without complications
UACR: Urinary albumin-to-creatinine ratio
WHR: Waist-hip ratio
