# Supplementary material for: Circulating levels of AGEs and soluble RAGE isoforms are associated with all-cause mortality and development of cardiovascular complications in type 2 diabetes: a retrospective cohort study
Source: Cardiovasc Diabetol. 2022 Jun 6;21:95. doi: 10.1186/s12933-022-01535-3 (PMC9169316; doi:10.1186/s12933-022-01535-3)
Supplement: Supplementary file 1 — Additional file 1: Table S1. Correlation matrix between selected clinical/biochemical variables and plasma levels of AGEs and sRAGE isoforms in CTR (n = 125) and T2DM (n = 362) subjects. Table S2. Binary logistic regression for the prediction of the development of MACE in patients with T2DM and no history of MACE at recruitment. [file 12933_2022_1535_MOESM1_ESM.pdf]

Supplementary Table 1. Correlation matrix between selected clinical/biochemical variables and plasma levels of AGEs and sRAGE isoforms in CTR (n=125) and T2DM (n=362) subjects.

		AGEs	sRAGE	esRAGE	cRAGE	AGE/sRAGE	AGE/esRAGE	AGE/cRAGE
AGEs	Spearman's rho	—						
	p-value	—						
sRAGE	Spearman's rho	0.080	—					
	p-value	0.068	—					
esRAGE	Spearman's rho	0.371***	0.440***	—				
	p-value	<.001	<.001	—				
cRAGE	Spearman's rho	-0.136**	0.762***	-0.152***	—			
	p-value	0.002	<.001	<.001	—			
AGE/sRAGE	Spearman's rho	0.863***	-0.393***	0.112*	-0.482***	—		
	p-value	<.001	<.001	0.011	<.001	—		
AGE/esRAGE	Spearman's rho	0.524***	-0.133**	-0.505***	0.163***	0.562***	—	
	p-value	<.001	0.002	<.001	<.001	<.001	—	
AGE/cRAGE	Spearman's rho	0.729***	-0.435***	0.316***	-0.728***	0.891***	0.264***	—
	p-value	<.001	<.001	<.001	<.001	<.001	<.001	—
Age	Spearman's rho	0.143**	-0.061	-0.044	-0.067	0.172***	0.206***	0.147***
	p-value	0.001	0.163	0.319	0.130	<.001	<.001	<.001
Disease duration	Spearman's rho	-0.039	0.004	-0.121*	0.076	-0.046	0.097	-0.079
	p-value	0.491	0.949	0.033	0.182	0.413	0.085	0.162
Weigth	Spearman's rho	0.115**	-0.031	-0.076	0.008	0.110*	0.194***	0.049
	p-value	0.009	0.482	0.085	0.861	0.012	<.001	0.263
BMI	Spearman's rho	0.113*	-0.030	-0.149***	0.036	0.107*	0.276***	0.039
	p-value	0.010	0.492	<.001	0.412	0.015	<.001	0.375
Waist/hip ratio	Spearman's rho	0.169***	-0.072	-0.147***	0.012	0.194***	0.300***	0.107*
	p-value	<.001	0.103	<.001	0.791	<.001	<.001	0.015
Glucose	Spearman's rho	0.305***	0.202***	-0.048	0.256***	0.165***	0.419***	0.007
	p-value	<.001	<.001	0.273	<.001	<.001	<.001	0.871
HbA1c	Spearman's rho	0.321***	0.211***	-0.053	0.250***	0.178***	0.437***	0.028
	p-value	<.001	<.001	0.229	<.001	<.001	<.001	0.526
Fasting insulin	Spearman's rho	0.068	-0.026	-0.128**	0.062	0.085	0.196***	0.008
	p-value	0.122	0.559	0.004	0.160	0.053	<.001	0.849
HOMA-index	Spearman's rho	0.188***	0.062	-0.136**	0.164***	0.142**	0.358***	0.009
	p-value	<.001	0.162	0.002	<.001	0.001	<.001	0.830
eGFR	Spearman's rho	-0.107*	-0.085	-0.045	-0.049	-0.048	-0.079	-0.046
	p-value	0.014	0.054	0.306	0.269	0.280	0.073	0.299
Uric acid	Spearman's rho	0.062	-0.039	-0.073	0.003	0.089*	0.107*	0.051
	p-value	0.156	0.377	0.096	0.948	0.043	0.015	0.248
AST	Spearman's rho	0.087*	-0.023	0.022	-0.039	0.087*	0.044	0.081
	p-value	0.047	0.596	0.621	0.381	0.049	0.318	0.065
ALT	Spearman's rho	0.162***	0.005	-0.072	0.054	0.138**	0.207***	0.056
	p-value	<.001	0.915	0.103	0.223	0.002	<.001	0.203
Hemoglobin	Spearman's rho	0.095*	-0.037	0.005	-0.019	0.110*	0.072	0.061
	p-value	0.031	0.398	0.918	0.672	0.012	0.103	0.167
Hematocrit	Spearman's rho	0.079	-0.084	-0.022	-0.059	0.125**	0.076	0.088*
	p-value	0.074	0.056	0.625	0.177	0.005	0.084	0.045
Monocyte %	Spearman's rho	0.019	-0.064	-0.042	-0.031	0.058	0.031	0.026
	p-value	0.659	0.144	0.347	0.478	0.193	0.482	0.549
Monocyte #	Spearman's rho	0.067	-0.049	-0.049	-0.003	0.080	0.083	0.038
	p-value	0.126	0.269	0.264	0.954	0.071	0.060	0.395
Transferrin	Spearman's rho	0.042	0.014	-0.057	0.065	0.049	0.095*	0.017
	p-value	0.341	0.745	0.196	0.144	0.273	0.031	0.708
Ferritin	Spearman's rho	0.094*	-0.077	-0.025	-0.033	0.114**	0.089*	0.047
	p-value	0.033	0.080	0.571	0.450	0.010	0.044	0.285

Note. * $p < .05$, ** $p < .01$, *** $p < .001$

Supplementary Table 1 (continued)

		AGEs_ugml	sRAGE_pgml	esRAGE	cRAGE	AGE_sRAGE_ratio	AGE_esRAGE_ratio	AGE_cRAGE_ratio
Total cholesterol	Spearman's rho	-0.042	-0.032	-0.030	-0.015	-0.022	-0.039	-0.006
	p-value	0.336	0.472	0.495	0.741	0.623	0.371	0.890
LDL-C	Spearman's rho	-0.097 *	-0.053	-0.069	-0.019	-0.054	-0.059	-0.029
	p-value	0.028	0.228	0.116	0.673	0.226	0.178	0.516
HDL-C	Spearman's rho	-0.084	-0.060	0.022	-0.090 *	-0.051	-0.128 **	0.007
	p-value	0.057	0.174	0.613	0.041	0.251	0.003	0.871
Tryglycerides	Spearman's rho	0.098 *	0.058	-0.045	0.084	0.057	0.167 ***	0.013
	p-value	0.026	0.187	0.307	0.057	0.197	<.001	0.772
ApoA1	Spearman's rho	-0.008	-0.089 *	0.063	-0.138 **	0.040	-0.100 *	0.092 *
	p-value	0.853	0.043	0.152	0.002	0.365	0.023	0.037
ApoB	Spearman's rho	-0.079	-0.062	-0.104 *	-0.006	-0.041	0.006	-0.036
	p-value	0.073	0.160	0.019	0.892	0.359	0.898	0.419
hs-CRP	Spearman's rho	0.172 ***	-0.038	-0.016	-0.035	0.162 ***	0.170 ***	0.130 **
	p-value	<.001	0.395	0.715	0.431	<.001	<.001	0.003
PAI-1	Spearman's rho	0.061	-0.071	-0.100 *	-0.033	0.104 *	0.163 ***	0.066
	p-value	0.169	0.107	0.023	0.459	0.018	<.001	0.132
Total protein	Spearman's rho	0.127 **	-0.068	-0.040	-0.032	0.160 ***	0.161 ***	0.118 **
	p-value	0.004	0.125	0.361	0.463	<.001	<.001	0.007
Telomere length	Spearman's rho	-0.154 ***	-0.019	-0.133 **	0.065	-0.119 **	-0.030	-0.132 **
	p-value	<.001	0.665	0.003	0.140	0.007	0.492	0.003
L-arginine	Spearman's rho	0.139 **	0.057	0.067	0.033	0.119 **	0.059	0.086
	p-value	0.002	0.203	0.135	0.460	0.008	0.189	0.053
ADMA	Spearman's rho	0.125 **	0.003	0.052	-0.031	0.131 **	0.071	0.098 *
	p-value	0.005	0.944	0.247	0.482	0.003	0.111	0.027
SDMA	Spearman's rho	0.077	-0.010	0.203 ***	-0.109 *	0.062	-0.137 **	0.112 *
	p-value	0.084	0.825	<.001	0.015	0.166	0.002	0.012

Note. * $p < .05$, ** $p < .01$, *** $p < .001$

Supplementary Table 2. Binary logistic regression for the prediction of the development of MACE in patients with T2DM and no history of MACE at recruitment.

	B	S.E.B.	Z	df	OR (95% CI)	p
Sex (male)	0.362	0.301	1.201	1	1.44 (0.80-2.59)	0.230
**Age (years)**	**0.045**	**0.021**	**2.176**	**1**	**1.05 (1.00-1.90)**	**0.030**
HbA1c (%)	0.089	0.121	0.737	1	1.10 (0.86-1.39)	0.461
hs-CRP (SD-increase)	0.008	0.022	0.361	1	1.01 (0.96-1.05)	0.718
Systolic blood pressure (10 mmHg increase)	0.0181	0.132	1.367	1	1.99 (0.92-1.55)	0.172
Metformin	0.166	0.230	0.558	1	1.18 (0.66-2.12)	0.577
Disease duration (years)	-0.010	0.012	-0.779	1	0.99 (0.96-1.01)	0.436
LDL-C (SD-increase)	0.196	0.147	-1.333	1	0.82 (0.61-1.10)	0.182
eGFR (10 mL/min increase)	0.002	0.007	0.258	1	1.00 (0.99-1.02)	0.797
**sRAGE (SD-increase)**	**0.394**	**0.147**	**2.688**	**1**	**1.48 (1.11-1.98)**	**0.007**

B, unstandardized regression coefficient; S.E. B, standard error of the coefficient; OR, odds ratio.
